# A tailored Internet-delivered modular intervention based on cognitive behavioral therapy for depressed older adults: a study protocol for a randomized controlled trial

**DOI:** 10.1186/s13063-021-05903-4

**Published:** 2021-12-16

**Authors:** Jonas Eimontas, Vilmantė Pakalniškienė, Ieva Biliunaite, Gerhard Andersson

**Affiliations:** 1 Institute of Psychology, Vilnius University, Vilnius, Lithuania; 2 Department of Behavioural Sciences and Learning, Department of Biomedical and Clinical Sciences, Linköping University, Linköping, Sweden; 3 Department of Clinical Neuroscience, Karolinska Institute, Stockholm, Sweden

**Keywords:** Depression, Elderly, CBT, ICBT, RCT, Internet-based intervention

## Abstract

**Background:**

Depression is most common among the elderly and is associated with major impairment. With limited accessible treatments available, remotely provided interventions are needed. Internet-based interventions have been proven effective for a number of mental and somatic health problems. However, the elderly population has received relatively limited attention in previous studies. This study aims to address this gap by investigating the effectiveness of a tailored Internet-delivered modular intervention based on cognitive behavioral therapy (CBT).

**Methods:**

A minimum of 60 participants will be recruited and randomly assigned to groups in a two-armed parallel controlled trial with a waiting list. The intervention group will have access to an 8-week therapist-supported modular intervention. The waiting list group will be instructed to wait for 8 weeks and then granted access to the intervention for 8 weeks. Pre, post, and 3-, 12-, and 24-month follow-up assessments are planned for measuring changes in depression symptoms, anxiety symptoms, and psychological well-being using PHQ-9, GDS, GAD-7, and WHO-5. Primary outcomes of all the participants will be analyzed using the intention-to-treat principle, and within- and between-group effect sizes will be calculated.

**Discussion:**

Internet-based interventions could help address the existing treatment gap for depressed older adults. However, to date, the effectiveness of Internet-based CBT (ICBT) for depressed older adults has only been tested in a few studies. This trial will demonstrate if Internet-based CBT is effective for this population when compared to a waiting list control. Further analysis of secondary outcomes and participant behavior in the intervention will potentially reveal effectiveness moderating factors.

**Trial registration:**

ClinicalTrials.gov NCT04728204. Registered on 15 January 2021. https://www.clinicaltrials.gov/ct2/show/NCT04728204?term=NCT04728204&draw=2&rank=1

## Administrative information

Note: the numbers in curly brackets in this protocol refer to SPIRIT checklist item numbers. The order of the items has been modified to group similar items (see http://www.equator-network.org/reporting-guidelines/spirit-2013-statement-defining-standard-protocol-items-for-clinical-trials/).

**Table:** 

Title {1}	A tailored internet-delivered modular intervention based on cognitive behavioral therapy for depressed older adults: a study protocol for a randomized controlled trial
Trial registration {2a and 2b}.	This study has been registered with clinicaltrials.gov with trial identifier number: NCT04728204 All items from the WHO Trial Registration Data Set were addressed during the registration.
Protocol version {3}	2021-01-24, version 1
Funding {4}	This project has received funding from the Research Council of Lithuania (LMTLT), agreement No 09.3.3-LMT-K-712-19-0192
Author details {5a}	Jonas Eimontas, PhD, Institute of Psychology, Vilnius University, Vilnius, Lithuania, jonas.eimontas@fsf.vu.lt (Corresponding author) Vilmantė Pakalniškienė, PhD, Institute of Psychology, Vilnius University, Vilnius, Lithuania, vilmante.pakalniskiene@fsf.vu.lt Ieva Biliunaite, MSc, Department of Behavioural Sciences and Learning, Linköping University, Linköping, ieva.biliunaite@liu.se Gerhard Andersson, PhD, Department of Behavioural Sciences and Learning, Linköping University, Linköping, Sweden; Department of Clinical Neuroscience, Karolinska Institute, Stockholm, Sweden, gerhard.andersson@liu.se
Name and contact information for the trial sponsor {5b}	Vilnius University, Universiteto 3, 01513, Vilnius, Lithuania. infor@cr.vu.lt
Role of sponsor {5c}	No sponsor has a part in the study design, data collection, management, analysis and interpretation, writing of the report or the decision to submit the report.

## Background

Depression is one of the most prevalent mental health disorders among the elderly [1, 2] and has been reported as one of the three largest contributors to the global burden of disease [3]. Studies have shown that depression can affect up to 8.1 % (major depressive disorder, MDD) and 14.1% and 24% (mild and sub-clinical depression) of the population [4]. Among individuals aged 65 to 100 years, 2.7% of men and 4.4% of women have major depression [5]. Lifetime prevalence of depression decreases with age and is estimated to be 9.6% for men and 20.4% for women [5]. Age itself has been demonstrated to have little to no effect on depression risk when controlling for income, physical disability, and social support [6]. Late-life depression has also been associated with cognitive dysfunction, disability, medical illnesses, and social isolation [7]. Finally, untreated depression can have serious implications on the quality and quantity of life [8].

Effective and accessible treatments are needed for treating depression in the elderly. A meta-analysis revealed that treating depressed older adults with antidepressants had unsatisfactory results and frequently led to adverse events [9]. Effective psychological treatments are therefore needed. A recent meta-analysis by Cuijpers and colleagues demonstrated that CBT and supportive counseling were effective for treating depression even after addressing biases [10]. However, because of barriers such as stigma, lack of providers and treatments, patient somatization, and denial, many cases of depression are not recognized and treated [11]. The global COVID-19 pandemic has limited the accessibility of face-to-face mental health services even further because older individuals are considered to be at higher risk and advised to limit their movement. Internet-based interventions have been developed and tested in controlled trials. These interventions could help fill this gap. Internet-based cognitive behavioral interventions have been found to be effective for a variety of mental and somatic health problems including depression when compared to their face-to-face equivalents [12, 13]. Most commonly depressed adult and to some extent adolescent populations have been the focus of previous research [14, 15]. To the best of our knowledge, only a few Internet-based CBT interventions for depressed older adults have been tested, but the ones that were tested report promising outcomes [16–18]. However, participant age was varied, with one trial including subjects as young as 50 years old [18]. Nevertheless, evidence on the long-term effects of such interventions is sparse and studies with longer follow-up periods [19] and older populations are needed.

Effectiveness is not the only concern with Internet-based CBT interventions. Providing treatment over the Internet poses dropout, recruitment and treatment tailoring challenges. Many previous Internet-delivered unguided treatments for depression and anxiety suffered from high dropout rates and adherence problems [20–22]. Existing evidence suggests that at least minimal therapist support improve adherence and treatment outcomes [23, 24]. In depression trials specifically, therapist assistance was associated with better outcomes and greater retention [25, 26]. Although therapeutic alliance seems to be high and somewhat similar to that documented in face-to-face treatments, it appears to have little relation to treatment outcomes [27]. Therapist guidance coupled with modular design allow for some level of individual tailoring and help avoid the one size fits all pitfall.

## Objectives {7}

The aim of this study is to test the effectiveness of an Internet-based cognitive behavioral therapy with minimal therapist support for depressed older adults. Our hypothesis is that the treatment group will have significantly larger reductions in symptoms of depression when compared to the waiting list control group post-treatment and will retain these effects at the 3-month follow-up. We will further seek to evaluate retention at 12- and 24-month follow-ups.

## Trial design {8}

This study is a parallel group, two-arm, exploratory trial with a 1:1 allocation ratio and no stratification.

## Methods

### Study setting {9}

This study will be managed from Vilnius University (Lithuania) and run in close collaboration with Linköping University (Sweden). The study will employ a secure and flexible platform developed and maintained by Linköping University [28]. Participants from all over Lithuania will be able to register as participants online. This will potentially provide an advantage in reaching a diverse demographic. The study will be conducted on a dedicated website.

### Eligibility criteria {10}

Inclusion criteria: Participants eligible for the trial must comply with all of the following at randomization: (1) 60+ years, (2) depressive symptoms, (3) access to a computer/tablet/smartphone and to the Internet, and (4) good knowledge of the Lithuanian language (reading, speaking, and writing without an interpreter). Exclusion criteria: (1) problematic alcohol use, (2) presently in any other psychological treatment, (3) severe depression, (4) suicidal ideation, and (5) significant change in psychiatric medication (during last 6 weeks).

### Who will take informed consent? {26a}

Individuals interested in participating in the study will have to visit the study website where they will receive information about the study. After familiarizing themselves with aims of the study and inclusion/exclusion criteria, they will then be redirected to a sub-site of the main website where they will be asked to read the informed consent form and to provide informed consent by selecting an option on the study website before any study procedures occur. The informed consent form is in Lithuanian language and describes the aims and procedures of the study in detail, the rights of the participants to withdraw the consent at any time and instructions to contact study team members in case of any study related adverse events during the trial. The consent form also provides contact details of the principle investigator for any study-related questions.

### Additional consent provisions for collection and use of participant data and biological specimens {26b}

Not applicable. There are no plans for additional studies using the data collected in this trial.

### Intervention

#### Explanation for the choice of comparators {6b}

In spite of the effectiveness of cognitive behavioral therapy provided online for depression, only a few trials have studied the applicability of ICBT for the elderly.

#### Intervention description {11a}

Eligible participants will be randomly assigned at a ratio of 1:1 either to a modular cognitive behavioral therapy group with minimal therapist support or a waiting list control group. Participants in the intervention group will receive access for 8 weeks to a tailored modular ICBT. Participants in the waiting list control group will be instructed to wait for 8 weeks before being able to access the intervention. This intervention was originally developed in Sweden and then translated and adapted for the Lithuanian population. A waiting list control group was chosen as a comparator because no other standardized Internet-based intervention for treatment of depression is available in Lithuanian language at this point in time.

All participants will receive the same first (introduction/rationale) and the last (relapse prevention) modules. The remaining modules will be tailored according to the participant’s condition and preferences. Tailoring will be done by selecting any 6 of the following 10 modules: behavioral activation (parts 1 and 2), acceptance, sleep disturbances, worry and anxiety, loneliness, pain, applied relaxation, the nature of emotions, and a life-review module. The intervention is constructed so that most modules begin with some psychoeducation, followed by exercises for participants to practice with and questions about the material presented in the module. Completed exercises and answered questions are then saved and can be assessed by the therapist. The therapists will assess the assignments, provide feedback, and prescribe modules weekly.

A group of three clinical psychologists and two graduate students of clinical psychology will be involved as therapists in this study. Supervision will be provided regularly by a clinical psychologist with experience in CBT and clinical work with patients suffering from anxiety and depression as well as Internet-delivered treatments.

#### Criteria for discontinuing or modifying allocated interventions {11b}

Participants will be instructed to contact the research team personnel if their condition deteriorates. Such a participant will be contacted within 24 h, and a thorough clinical interview will be administered via telephone by an experienced clinical psychologist. Based on the outcome of the interview, the participant might then be referred to a hospital or other appropriate services.

#### Strategies to improve adherence to interventions {11c}

Adherence to the intervention will be monitored through the treatment platform. The platform automatically records participant engagement with the intervention. Recorded activity includes login times, module completion, exercise completion, and number of messages sent to the therapist.

#### Relevant concomitant care permitted or prohibited during the trial {11d}

Ongoing psychopharmacological medications with stable dosage are allowed. Participation in other psychological interventions is prohibited but will not result in withdrawal from the study if started after the intervention. After termination, all participants will be asked about receiving any other intervention during the trial and this information will be reported.

#### Provisions for post-trial care {30}

Participants who show signs of deterioration will be directed to their local mental health center for further treatment.

### Outcomes {12}

Difference between intervention and control groups pre- and post-treatment as well as at 3-month and 1- and 2-year follow-ups will be analyzed for both primary and secondary outcomes. Primary outcomes are depression symptoms measured with the Patient Health Questionnaire (PHQ-9) [29] and Geriatric Depression Scale (GDS) [30]. Secondary outcomes include anxiety symptoms and psychological well-being. Anxiety symptoms will be measured with the Generalized Anxiety Disorder Scale (GAD-7) [31] and levels of psychological well-being will be measured with the World Health Organization Well-being Index (WHO-5) [32]. Difference in means post-treatment will be compared between groups for both primary and secondary outcomes. Within group trial effects will be calculated using difference in means from baseline to post-treatment for all primary and secondary outcomes. Additionally long-term effectiveness of the intervention will be measured comparing outcome differences from post-treatment to 3-month and 1- and 2-year follow-ups.

### Participant timeline {13}

Participant timeline is shown in Table 1.

**Table 1:** Schedule of enrolment, interventions, and assessments

Assessment/activity	Online screening/baseline	Telephone interview	Post-randomization		Follow-up		
					***f1***	***f2***	***f3***
	***−t***_***1***_	***t0***	***t***_***1***_***–t***_***8***_ (weekly)	***t***_***9***_ (post)			
Informed consent	X						
Demographic data	X						
ICBT			X				
PHQ-9	X			X	X	X	X
GDS	X			X	X	X	X
GAD-7	X			X	X	X	X
WHO-5	X			X	X	X	X
AUDIT	X			X	X	X	X
M.I.N.I (Depression)		X					
Treatment expectancy		X					
Treatment engagement			X				
NEQ				X			

*M.I.N.I (Depression)* Mini International Neuropsychiatric Interview Depression part, *ICBT* Internet-based cognitive behavior therapy, *GAD-7* Generalized Anxiety Disorder-7, *PHQ-9* Patient Health Questionnaire, *GDS* Geriatric Depression Scale, *WHO-5* World Health Organization Well-Being Index, *AUDIT* Alcohol Use Disorders Identification Test, *F*_*1*_ 3 months, *F*_*2*_ 12 months, *F*_*3*_ 24 months

### Sample size {14}

A power calculation was conducted using GLIMMPLSE software, which is helpful for sample-size calculation for repeated measures and longitudinal studies [33]. Statistical power was set to 0.8, including pre- and post-treatment repeated measurement points; an *α* was set at 0.05. A total sample of 70 participants was needed to reach 80% power. Factoring in an estimated dropout rate of 10% we seek to randomize 78 participants (39 participants in each group). The power analysis is based on the primary outcome variable’s Patient Health Questionnaire (PHQ-9) [29] means and standard deviations of the two time points data based on the results from a similar unpublished study from Sweden (intervention group pre-treatment (T1) *M* = 12.45, *SD* = 4.38, post-treatment (T2) *M* = 6.57, *SD* = 4.34, control group T1 *M* = 14.22, *SD* = 4.19, T2 *M* = 8.11 *SD* = 4.92), with a 1:1 allocation ratio.

### Recruitment {15}

Participants will be recruited through social media outlets, a press release and advertisement in local papers, interviews on radio, and local television. In order to obtain a needed sample size, recruitment period might be prolonged and paid advertisement in social media targeting older adults might be intensified.

## Assignment of interventions: allocation

### Sequence generation {16a}

An independent researcher will use a random number generator (random.org) to randomly assign all participants at a 1:1 ratio to study groups without stratification.

### Concealment mechanism {16b}

No concealment will be used because it is not possible due to the nature of the trial design.

### Implementation {16c}

**Fig. 1:**
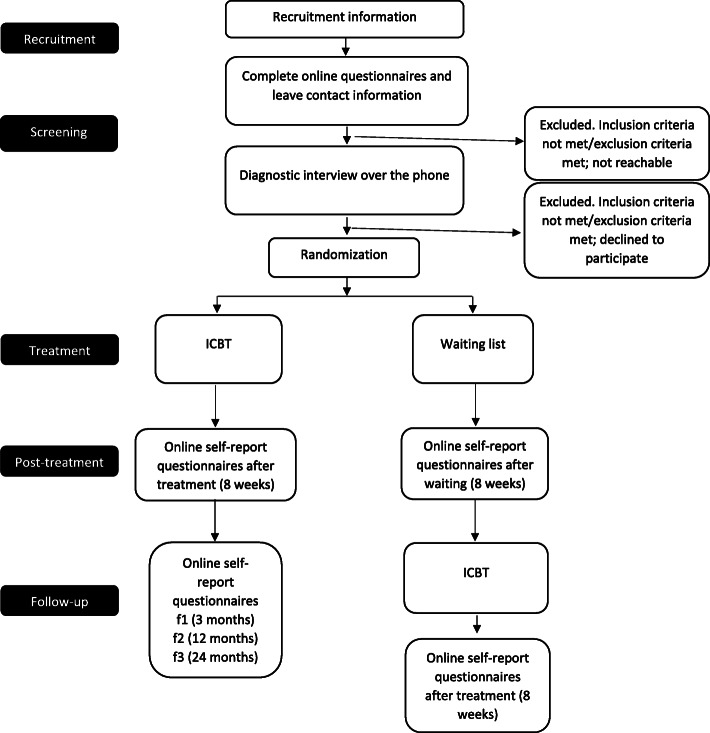
CONSORT diagram

All participants will be self-referred. Participants will be enrolled and assigned to trial groups by different study team members (Fig. 1).

## Assignment of interventions: blinding

### Who will be blinded {17a}

Due to the nature of the psychological intervention no one from the participants and study team members will be blinded after assignment to intervention. Only data analysts will be blinded to the study groups until all data analyses are completed.

### Procedure for unblinding if needed {17b}

Not applicable because no one will be blinded.

## Data collection and management

### Plans for assessment and collection of outcomes {18a}

Data will be collected at pre-randomization, termination, and 3-, 12-, and 24-month follow-ups. All questionnaires will be self-report and administered online. To ensure collected data is complete and accurate, the online questionnaires will be coded in a way that requires participants to fully answer the questions in order to submit the answers. For a full timeline of questionnaire dissemination, see Table 1.

#### Primary outcomes

Depression symptoms will be measured with the Patient Health Questionnaire (PHQ-9) [29] and Geriatric Depression Scale (GDS) [30]. On the PHQ-9, total scores range from 0 to 27, and a score of four or less indicates no depression, while scores from 5 to 9 points are considered to reflect sub-clinical depression. On the GDS, scores can range between 0 and 15. A score of five or less is considered within the normal range.

#### Secondary outcomes

In addition to symptoms of depression, symptoms of anxiety and general well-being will be measured. The Generalized Anxiety Scale (GAD-7) [31] is a 7-item scale with total scores ranging from 0 to 21. Scores above 10 warrant further investigation of generalized anxiety disorder. The World Health Organization Well-being Index [32] is a 5-item questionnaire. Respondents are asked to rate how they felt during the past two weeks by choosing the best corresponding answer using a 6-item Likert scale where a score of 0 indicates ‘At no time’ and a score of 5—‘All the time’. A higher score indicates greater well-being.

### Plans to promote participant retention and complete follow-up {18b}

All participants will be informed in the informed consent about follow-up assessments. If participants discontinue or deviate from intervention protocols, they will be contacted and study team members will first try to address any concerns participants might have that are altering their adherence to the intervention protocols. In case these concerns cannot be resolved, participants will then be asked to fill in follow-up self-assessment questionnaires online.

### Data management {19}

Most of the data gathered online and saved to a database will be coded automatically. Only a small fraction of demographic data will need to be coded. Data will be stored on a secure server and, after the termination of the study, uploaded to MIDAS, a secure national scientific data repository, where it will be available upon request to corresponding author after reports are published.

### Confidentiality {27}

After giving informed consent, potential and enrolled participants will be asked to provide their email address and telephone number. The platform generates a unique code for each registered individual and stores it on a secure server available only to the research team with administrative rights after logging. Only non-personal data will be available from the data repository after the trial.

### Plans for collection, laboratory evaluation and storage of biological specimens for genetic or molecular analysis in this trial/future use {33}

Not applicable, no samples collected.

## Statistical methods

### Statistical methods for primary and secondary outcomes {20a}

Effect sizes for pre- to post-treatment and between-group comparisons for the main and secondary outcomes will be calculated to evaluate the effectiveness of the intervention. Additionally, regression analysis will be performed to test if treatment expectations prior to the beginning of the treatment predict primary and secondary outcomes.

### Interim analyses {21b}

No interim analysis is planned in this trial.

### Methods for additional analyses (e.g., subgroup analyses) {20b}

Not applicable, subgroup analyses are not planned.

### Methods in analysis to handle protocol non-adherence and any statistical methods to handle missing data {20c}

Initially, intention-to-treat (ITT) analysis will be performed on all primary outcomes in case of drop-out from the study. Secondary per protocol analyses (PPA) will be performed for those participants who adhered to treatment protocol. In ITT analysis last value carried forward will be used to fill in missing data. In PPA analysis missing cases will be removed on a case-by-case basis and only completed data will be analyzed.

### Plans to give access to the full protocol, participant-level data, and statistical code {31c}

Depersonalized data will be archived and available upon request to PI from the Lithuanian research data storing platform midas.lt.

## Oversight and monitoring

### Composition of the coordinating center and trial steering committee {5d}

Not applicable, no coordinating center or trial steering committee was planned for this trial. This is a trial of a psychosocial intervention with one core study team solely responsible for coordination of the trial. Most data collection is automated and all actions with the database are recorded ensuring integrity of the database. Any significant amendments to study protocol will be provided to and verified by the Research Ethics Committee. An independent physician with training in psychiatry and experience in clinical work will be available for consultation during the trial.

### Composition of the data monitoring committee, its role, and reporting structure {21a}

A data monitoring committee is not necessary because all participants will be self-referred, all data will be coded directly to the system where no adjustments can be made to data, and all actions are logged. Furthermore, participants will have weekly contact with research team members who will monitor for any adverse events and report them to principle investigator. Participants who experience adverse events will be interviewed by a study team member with experience in clinical assessment and will be informed about available community services appropriate for their condition.

### Adverse event reporting and harms {22}

During the trial, enrolled participants will be informed to report adverse effects to the trial administrator. An experienced clinician from the research team will contact any such participants and conduct a thorough investigation. Additionally, although adverse events in interventions are rare, post-treatment assessment will be conducted as has been recommended by Rozental and colleagues [34]. All participants initially in the intervention group and, after receiving treatment, the waiting list group will be asked to complete the Negative Effects Questionnaire (NEQ) to record any negative trial effects. All gathered data on harms will be published together with main outcome results.

### Frequency and plans for auditing trial conduct {23}

Vilnius University does not have a dedicated research audit department.

### Plans for communicating important protocol amendments to relevant parties (e.g., trial participants, ethical committees) {25}

Any amendments to study protocol would first have to be approved by the Vilnius University Research Ethics Committee. If approved, these changes would then have to be reported in the trial register. Finally, any amendments would be reported in the final report of research data.

## Dissemination plans {31a}

Trial results will be published in a peer-reviewed journal. The results will also be presented in scientific conferences and a popular science paper will be prepared. A public engagement event will be organized to present trial results to the public. After the publication of trial results, an anonymized data set will be published in the Lithuanian research data repository MIDAS and available upon request to the PI.

## Discussion

There was already an urgent need for effective remotely delivered psychosocial interventions for the elderly but the global COVID-19 pandemic has brought this urgency to new heights [35]. Internet-based interventions could help reduce barriers associated with access to mental health care [23]. However, little is known about the effectiveness of Internet-based interventions for depressed older adults. This study aims to address this gap by testing the effectiveness of an Internet-based modular CBT intervention for depressed older adults. We expect treatment to significantly reduce symptoms of depression and anxiety. We also expect the intervention to be superior in this regard to the control waiting list group by a clinically significant margin. The strength of this study is its modular format and we will therefore be able to assess how the prescribed modules interact with effectiveness.

A potential limitation of this trial is the use of a waiting list as a control group [10, 36]. Waiting list control groups have been reported to inflate effect sizes in between-group comparisons. One possible limitation could also stem from participants being self-referred as this might limit the generalizability of the results. However, a recent study on recruitment strategies for Internet-based interventions found that participants recruited through Google were comparable in symptoms to those recruited from clinical settings [37]. Our study might also shed more light on factors associated with effectiveness and retention. With populations in high-income countries aging, it is important to understand which intervention factors contribute to the effectiveness of Internet-based treatment as well as what physical, psychological, and social factors related to aging add to the equation.

## Trial status

2021-01-24, Protocol version 1. Recruitment commenced on January 28, 2021, and is planned to extend to May 2021. It was not possible to publish the protocol before recruitment because funding for the study was approved 2 months later than announced. These circumstances caused the study team to speed up preparations to catch up to the original timeline so that the recruitment could begin on time.

## Data Availability

After the publication of trial results, an anonymized data set with will be uploaded to the Lithuanian research data repository MIDAS and available upon request from the PI.

## Abbreviations

CBT: Cognitive behavioral therapy
CONSORT: Consolidated standards of reporting trials
ICBT: Internet-based cognitive behavioral therapy
ITT: Intention to treat
MDD: Major depressive disorder
PI: Principal investigator
PPA: Per protocol analysis
RCT: Randomized controlled trial
